# Effect of different sources and inclusion levels of dietary fat on productive performance and egg quality in laying hens raised under hot environmental conditions

**DOI:** 10.5713/ajas.19.0063

**Published:** 2019-04-15

**Authors:** Jong Hyuk Kim, Han Kyu Lee, Tae Sung Yang, Hwan Ku Kang, Dong Yong Kil

**Affiliations:** 1 Department of Animal Science and Technology, Chung-Ang University, Anseong 17546, Korea; 2 Poultry Research Institute, National Institute of Animal Science, Rural Development Administration, Pyeongchang 25342, Korea

**Keywords:** Egg Quality, Fat Source, Hot Environmental Condition, Inclusion Level of Dietary Fat, Laying Hen, Productive Performance

## Abstract

**Objective:**

This experiment aimed to investigate the effect of different sources and inclusion levels of dietary fat on productive performance and egg quality in laying hens raised under hot environmental conditions.

**Methods:**

A total of 480 Hy-Line Brown laying hens at 31 wk of age were randomly allotted to 1 of 5 experimental diets. The control diet contained 2,800 kcal/kg nitrogen-corrected apparent metabolizable energy with no fat addition. Four additional diets were prepared by adding 2.0% or 4.0% of animal fat (AF) or soybean oil (SO). Energy and nutrient concentrations were consistent among all diets. Diets were fed to hens for 4 weeks. Average daily room temperature and humidity were 26.7°C±1.52°C and 77.4%±4.50%. The heat stress index was approximately 76, indicating that hens were raised under heat stress conditions.

**Results:**

Final body weight (BW) was greater (p<0.05) for hens fed diets containing 2.0% or 4.0% AF than for those fed the control diet or diets containing 2.0% or 4.0% SO. The BW gain and feed intake were greater (p<0.05) for hens fed diets containing additional AF or SO than those fed the control diet. Eggshell thickness was the greatest (p<0.05) for hens fed the control diet, but the least (p<0.05) for hens fed diets containing 4.0% SO. Egg yolk color was the greatest (p<0.05) for hens fed the control diet, but the least (p<0.05) for hens fed diets containing 4.0% SO.

**Conclusion:**

Inclusion of supplemental fat (AF and SO) in diets exhibits preventative effects on BW loss for hens raised under hot environmental conditions when energy and nutrient concentrations in diets were maintained. The effects were greater for AF than for SO. However, inclusion of supplemental fat in diets decreases eggshell thickness and egg yolk yellowness, possibly due to a reduction in Ca absorption and intake of egg yolk colorants.

## INTRODUCTION

Hot environmental conditions exert a negative effect on the productive performance and welfare of poultry [1,2]. In laying hens, continuous exposures to hot environmental conditions can lead to hyperthermia, which is typically associated with poor productive performance and egg quality [3]. Various management strategies including dietary treatments have been developed to ameliorate the negative effects of hot environmental conditions on laying hens. However, no promising practices have been identified.

Ambient temperature is one of the main factors affecting energy requirements because animals increase their energy use for releasing heat from the body in various ways, such as radiation and panting [4]. In addition, animals exposed to heat stress generally attempt to reduce their feed intake (FI) as a natural defense mechanism against increasing heat loads because FI itself inevitably results in heat production from the body [4,5]. As a result, animals exposed to heat stress often suffer from a marginal deficiency of energy and nutrients. To prevent this deficiency, energy and nutrient concentrations in diets are typically increased in a commercial situation. In addition, other procedures to promote FI or inclusion of functional additives in diets are also adopted to ameliorate heat stress in animals [5–7].

Dietary fat has been widely used to increase energy con centrations in diets for animals exposed to heat stress [8–10]. In addition, dietary fat has an additional benefit called “extra-caloric effect” [11]. This extra-caloric effect of dietary fat is known to result from increased retention time of digesta in the gastrointestinal tract (GIT), which increases nutrient utilization in diets [11–13]. Finally, dietary fat induces less heat production during metabolism (i.e., heat increment) than other nutrients [14], and therefore, decreases heat loads for animals raised under hot environmental conditions [4,15,16]. Therefore, previous studies have shown that inclusion of supplemental fat in diets improved productivity of broiler chickens raised under hot environmental conditions [10,17]. However, the data regarding the effect of different sources and levels of supplemental fat in diets with the constant levels of energy and nutrients on laying hens are scarcely available.

Therefore, the objective of the current experiment was to investigate the effect of different sources and inclusion levels of dietary fat on productive performance and egg quality of laying hens raised under hot environmental conditions.

## MATERIALS AND METHODS

### Animals, diets, and experimental design

The protocol for the current experiment was reviewed and approved by the Institutional Animal Care and Use Committee at Chung-Ang University. A total of 480 Hy-Line Brown laying hens at 31 wk of age were distributed in a completely randomized design to 1 of 5 dietary treatments with 8 replicates. Each replicate had 6 consecutive cages with 2 hens per cage. Hens were raised during the hot season in the Republic of Korea (from the end of July to the end of August). The average daily maximum and minimum ambient temperatures were 29.9°C and 23.6°C, respectively. The overall averages of ambient temperature and relative humidity were 26.7°C± 1.52°C and 77.4%±4.50%, respectively. Based on heat stress index calculated from the ambient temperature and relative humidity for commercial laying hens [18], heat stress index in our environmental conditions was approximately 76, which indicated that laying hens in the current experiment were raised under severe heat stress conditions [18]. A commercial-type control diet was prepared to meet or exceed NRC [19] requirement estimates for laying hens (Table 1). Four additional diets were prepared by adding 2.0% or 4.0% of either animal fat (AF) or soybean oil (SO) to the control diet at the expense of other ingredients such as corn, soybean meal, and corn gluten meal. Both fat sources were obtained from a commercial feed company. The diets containing supplemental fat were formulated with energy and nutrient concentrations that were identical to those of the control diet in order to avoid the potential effect of different energy and nutrient concentrations among dietary treatments. A 16-h lighting schedule was applied throughout the experiment. Diets were fed to hens on *ad libitum* basis for 4 weeks.

### Data collection and sample analysis

Initial body weight (BW), final BW, BW gain, hen-day egg production, egg weight, egg mass, FI, and feed conversion ratio (FCR) were recorded and calculated [20]. The data for productive performance were summarized on 4 wk. Egg quality was assessed with four eggs per replicate randomly collected at the conclusion of the experiment. Egg quality was measured by the method described by Kim et al [20]. Briefly, eggshell strength was determined using a texture analyzer (model TAHDi 500, Stable Micro System, Godalming, UK) and was displayed as unit of compression force exposed to the unit eggshell surface area. Eggshell thickness was measured at three different positions of eggshells with a dial pipe gauge (model 7360, Mitutoyo Co., Ltd., Kawasaki, Japan). Eggshell color was determined using the eggshell color fan (Samyangsa, Kangwon, Korea; where 15 = very dark brown and 1 = very light and pale). Egg yolk color was estimated by the Roche color fan (Hoffman-La Roche Ltd., Basel, Switzerland; where 15 = dark orange and 1 = light pale). Haugh unit was determined using the equations as described by Eisen et al [21].

### Statistical analysis

All data were analyzed by analysis of variance as a completely randomized design using the PROC MIXED procedure of SAS (SAS Institute, Inc., Cary, NC, USA). Data were checked for outliers using the UNIVARIATE procedure of SAS [22], but no outliers were identified. The replicate was used as an experimental unit in all analyses. Dietary treatment was included as a fixed effect in the model and the LSMEANS procedure was used to calculate treatment means if the difference was significant [23]. Means were separated by the PDIFF option in SAS. Significance for statistical tests was set at p<0.05.

## RESULTS

Hens fed diets containing 2.0% or 4.0% AF had a greater (p< 0.05) final BW than those fed the control diet or diets containing 2.0% or 4.0% SO (Table 2). The BW gain for hens fed diets containing 2.0% or 4.0% AF or diets containing 4.0% SO was greater (p<0.05) than for those fed the control diet. During 4-week feeding trial, hens fed the control diet exhibited the greater loss of BW, which was not the case for hens fed diets containing supplemental fat. However, hen-day egg production, egg weight, egg mass, and FCR were not affected by inclusion of supplemental fat in diets. Interestingly, hens fed diets containing supplemental fat had a greater (p<0.05) FI than those fed the control diet, despite the fact that dietary energy concentrations were consistent among dietary treatments.

The eggshell thickness was less (p <0.05) for hens fed diets containing 4.0% SO than for those fed the control diet (Table 3). Likewise, eggshell strength was also the least for hens fed diets containing 4.0% SO although significance was not detected. A decrease (p<0.05) in egg yolk color by feeding diets containing supplemental fat was observed with more fat addition resulting in less egg yolk yellowness. Hens fed diets containing 4.0% SO exhibited less (p<0.05) egg yolk color scores than those fed diets containing 4.0% AF.

## DISCUSSION

The observation that hens fed the control diet had high BW loss without changes in egg production indicates that hens raised under hot environmental conditions lose body energy and nutrient storages. This is likely due to increased energy and nutrient requirements but decreased FI. In addition, our results further indicate that laying hens exposed to heat stress likely prioritize egg production over body maintenance. Similar results were also observed in laying hens previously [9]. To overcome this negative effect of hot environmental conditions, increasing energy and nutrient concentrations in diets by inclusion of supplemental fat or other nutrients has been widely practiced in a commercial situation [24–26]. In the current experiment, likewise, inclusion of 2.0% or 4.0% supplemental fat in diets prevented hens from losing their BW, despite the fact that energy and nutrient concentrations were consistent among dietary treatments. The reason for this positive effect is likely related to an increase in FI by feeding hens diets containing supplemental fat as also was observed in this experiment. It is known that dietary fat produces the least amounts of heat during metabolism (i.e., heat increment) among 3 energy-yielding nutrients [14]. Therefore, energy supplies from fat in diets in replace of carbohydrates and proteins can reduce heat loads for animals raised under hot environmental conditions [27]. This is the reason why we observed increased FI in hens fed diets containing supplemental fat, which subsequently prevented BW loss of hens. In addition, inclusion of supplemental fat in diets may aid in increasing nutrient utilization in diets by increasing retention time of digesta in the GIT, which is called the extra-caloric effect of dietary fat [11–13], supporting increased needs for energy and nutrients in laying hens raised under hot environmental conditions.

Interestingly, inclusion of AF resulted in more preventa tive effects on BW loss of hens than inclusion of SO although FI was not influenced by dietary fat sources. The reason for this observation is likely related to differences in fat metabolism between these 2 fat sources and subsequently their net energy (NE) values. Kil et al [28] reported that AF had a greater NE value than SO in pig diets because SO contains greater amounts of unsaturated fatty acids, which may increase energy requirements for maintenance. This is possibly due to increased oxidative stress resulting from SO as compared to AF high in saturated fatty acids. Similar results of better performance of young pigs were observed when pigs were fed diets containing lard than when they were fed diets containing SO [29]. However, there was no additional benefit of increasing inclusion levels from 2.0% to 4.0% of both AF and SO on productive performance of laying hens raised under hot environmental conditions. This result was not expected because inclusion of more fat in diets decreases heat loads of hens to a greater extent, which leads to a greater benefit on laying hens raised under hot environmental conditions. Therefore, the reason for this result is not clear; however, it is may related to the fact that possible negative effects of high fat inclusion in diets such as decreasing mineral utilization offset additional benefits from increasing inclusion from 2.0% to 4.0% supplemental fat in diets [30–32]. Kil et al [28] also observed with unknown reason that NE of diets were not influenced by increasing inclusion levels of dietary fat from 5.0% to 10.0% SO in diets fed to pigs.

Inclusion of supplemental fat in diets decreased eggshell thickness in laying hens although the significant reduction was observed only for hens fed diets containing 4.0% SO. It was expected that increasing FI by inclusion of supplemental fat would have a beneficial effect on eggshell thickness because of increasing intake of essential minerals for eggshell formation such as Ca and P. However, we found that inclusion of supplemental fat in diets resulted in decreased eggshell thickness. Therefore, we speculate that inclusion of supplemental fat in diets may exert a negative effect on mineral utilization (e.g., digestion and absorption), especially for Ca. It has been reported that increasing inclusion of dietary fat increased soap formation of Ca with fatty acids in the GIT, which ultimately decreases Ca absorption for animals [30–32]. However, it is not clear why eggshells were significantly thinner only for hens fed diets containing 4.0% SO. Differences in the extent of Ca soap formation between AF and SO may be the reason because soap formation of Ca with fatty acids may be affected by the characteristics of fatty acids such as chain length and the number of double bonds in fatty acids [33,34]. However, we observed that eggshell strength was not affected by inclusion of supplemental fat in diets although eggshell thickness differed among dietary treatments.

The extent of yellowness in egg yolk color is known to be largely dependent on the concentration and type of dietary colorants (e.g., xanthophyll) [35,36]. Therefore, our observation for decreased egg yolk color by feeding diets containing supplemental fat is likely a consequence of replacing corn grains and corn gluten meal, which contain high amounts of egg yolk colorants, with supplemental fat. Shin et al [36] reported that decreasing inclusion of distillers dried grains with solubles and corn gluten meal in diets linearly decreased egg yolk color of laying hens. Similarly, we found that increasing inclusion levels of dietary fat induced a further reduction in egg yolk color. This observation also explains why we found that hens fed diets containing 4.0% SO had less egg yolk color than those fed diets containing 4.0% AF as more corn grains and corn gluten meal were replaced by inclusion of 4.0% SO than by inclusion of 4.0% AF. These results suggest that inclusion of supplemental fat in the replacement of feed ingredients high in egg yolk colorants inevitably decreases yellowness of egg yolk color in laying hens if no additional colorants are included in diets.

## CONCLUSION

Inclusion of supplemental fat (AF and SO) has a preventative effect on BW loss in laying hens raised under hot environmental conditions when energy and nutrient concentrations in diets were maintained. However, inclusion of more than 2.0% supplemental fat, regardless of fat sources, exerts no further beneficial effects. Inclusion of AF results in more positive effects on the prevention of BW loss in laying hens than inclusion of SO. However, inclusion of supplemental fat in diets adversely affects eggshell thickness and egg yolk coloration, possibly due to impaired Ca absorption and reduced intake of egg yolk colorants.

## Figures and Tables

**Table 1:** Ingredient composition of experimental diets

Item (%, unless noted)	Control	Animal fat1)		Soybean oil1)	
	
		2.0%	4.0%	2.0%	4.0%
Ingredient					
Corn grains	65.80	61.65	57.49	61.27	56.73
Animal fat	-	2.00	4.00	-	-
Soybean oil	-	-	-	2.00	4.00
Soybean meal	7.40	14.96	22.53	16.19	24.98
Corn gluten meal	11.76	6.76	1.75	5.90	0.05
Monodicalcium phosphate	1.27	1.25	1.24	1.25	1.23
Limestone	10.71	10.66	10.61	10.65	10.59
Salt	0.29	0.30	0.30	0.31	0.33
L-lysine (H_2_SO_4_)	0.69	0.44	0.20	0.40	0.12
DL-methionine	0.13	0.19	0.24	0.19	0.25
L-threonine	0.07	0.06	0.05	0.06	0.04
L-tryptophan	0.47	0.31	0.15	0.31	0.15
Choline chloride (50%)	0.05	0.05	0.05	0.05	0.05
NaHCO_3_	0.10	0.11	0.13	0.16	0.22
Vitamin premix2)	0.13	0.13	0.13	0.13	0.13
Mineral premix2)	0.13	0.13	0.13	0.13	0.13
Celite	1.00	1.00	1.00	1.00	1.00
Total	100.00	100.00	100.00	100.00	100.00
Energy and nutrient contents3)					
AME_n_ (kcal/kg)	2,800	2,800	2,800	2,800	2,800
Crude protein (%)	16.00	16.00	16.00	16.00	16.00
Crude fat (%)	2.57	4.46	6.36	4.45	6.34
Calcium (%)	4.20	4.20	4.20	4.20	4.20
Available P (%)	0.33	0.33	0.33	0.33	0.33
Lysine (%)	0.85	0.87	0.89	0.87	0.89
Cys+Met (%)	0.73	0.73	0.74	0.74	0.74
Digest. Lys (%)	0.79	0.79	0.79	0.79	0.79
Digest. Cys+Met (%)	0.67	0.67	0.67	0.67	0.67
Linoleic acid (%)	1.20	1.31	1.41	2.15	3.10

AME_n_, nitrogen-corrected apparent metabolizable energy.

1) Animal fat and soybean oil were obtained from a commercial feed company.

2) Provided per kilogram of the complete diet: vitamin A, 12,500 IU; vitamin D_3_, 2,500 IU; vitamin E 20 IU; vitamin K_3_, 2 mg; vitamin B_1_, 2 mg; vitamin B_2_, 5 mg; vitamin B_6_, 3 mg; vitamin B_12_, 18 μg; folic acid, 1 mg; biotin 50 μg; niacin, 24 mg; zinc, 60 mg; manganese 50 mg; iron, 50 mg; copper, 6 mg; cobalt, 250 μg; iodine, 1 mg; selenium, 150 μg.

3) Calculated values from NRC [19].

**Table 2:** Effect of different sources and inclusion levels of dietary fat on productive performance of laying hens raised under hot environmental conditions1)

Items	Control	Animal fat		Soybean oil		SEM	p-value
	
		2.0%	4.0%	2.0%	4.0%		
Initial BW (g)	2,048	2,102	2,078	2,074	2,065	16.1	0.25
Final BW (g)	1,999^b^	2,135^a^	2,114^a^	2,072^b^	2,076^b^	15.8	<0.01
BW gain (g)	−49^b^	33^a^	36^a^	−1^ab^	11^a^	13.6	<0.01
Hen-day egg production (%)	92.5	93.2	93.7	94.3	93.5	1.24	0.89
Egg weight (g)	62.8	63.7	63.5	63.6	64.2	0.49	0.33
Egg mass (g)	58.1	59.4	59.6	61.0	60.0	0.88	0.33
Feed intake (g/d)	104^b^	109^a^	110^a^	108^a^	109^a^	1.37	0.02
FCR (g/g)	1.78	1.84	1.84	1.79	1.81	0.02	0.22

SEM, standard error of the mean; BW, body weight; FCR, feed conversion ratio.

1) Each value represents the mean of 8 replicates per treatment.

a,b Mean values with different superscripts have significant difference (p<0.05).

**Table 3:** Effect of different sources and inclusion levels of dietary fat on egg quality of laying hens raised under hot environmental conditions1)

Items	Control	Animal fat		Soybean oil		SEM	p-value
	
		2.0%	4.0%	2.0%	4.0%		
Eggshell strength (kg/cm^2^)	3.21	3.23	3.12	3.21	2.78	0.14	0.12
Eggshell thickness (μm)	413^a^	398^ab^	400^ab^	403^ab^	393^b^	3.80	0.01
Eggshell color2) (color fan)	14.5	13.6	14.2	13.7	14.1	0.28	0.12
Egg yolk color3) (color fan)	8.8^a^	8.5^b^	6.6^c^	7.8^b^	5.5^d^	0.11	0.01
Haugh unit4)	98.1	98.1	95.2	95.6	96.0	1.21	0.28

SEM, standard error of the mean.

1) Each value represents the mean of 8 replicates per treatment.

2) Eggshell color was evaluated by the eggshell color fan (15 = dark brown and 1 = light white).

3) Egg yolk was evaluated by the Roche color fan (15 = dark orange and 1 = light pale).

4) Haugh unit was calculated based on the equation (H, albumen height; W, egg weight): Haugh unit = 100 log (H–1.7W^0.37^+7.6).

a–d Mean values with different superscripts have significant difference (p<0.05).
